# A cavernous hemangioma of infundibulopelvic vessels presenting as an adnexal tumor: A rare case report and literature review

**DOI:** 10.1097/MD.0000000000030113

**Published:** 2022-08-19

**Authors:** Hongwei Ma, Huiyun Tang, Qian Chen, Wen Zheng, Xin Tan

**Affiliations:** a Department of Obstetrics·and Gynecology, Key Laboratory of Birth Defects and Related Diseases of Women and Children, Ministry of Education, West China Second University Hospital, Sichuan University, No. 20, Section 3, Renmin Nanlu, Chengdu, 610041, China; b Laboratory of Clinical Proteomics and Metabolomics, Institutes for Systems Genetics, Frontiers Science Center for Disease-related Molecular Network, West China Hospital, Sichuan University, 88 Keyuan South Road, Hi-Tech Zone, Chengdu, 610041, China.

**Keywords:** adnexal mass, cavernous hemangiomas, infundibulopelvic vessels

## Abstract

**Rationale:**

Female reproductive organ angiomas are rarely reported and are accidentally found during surgery. Angiomas arising from infundibulopelvic vessels presenting as adnexal masses are even rarer, and a few doctors have experience in their management.

**Patient’s main concerns and important clinical findings:**

Herein, we report the case of a 40-year-old woman who was admitted after a physical examination revealed an ovarian mass. The physical examination revealed a palpable adnexal mass in the right pelvic cavity. Ultrasound showed a 4.5 × 4.0 × 5.0 cm space-occupying lesion close to the right ovary, which had many echogenic lines and calcifications in its cystic cavity.

**Primary diagnosis:**

Right adnexal mass.

**Interventions:**

Laparoscopic surgery was performed in all the patients. During the surgery, the mass was found to be a retroperitoneal hemangioma with distorted and dilated vessels. We separated the right infundibulopelvic vessels and performed tumor resection with minimal blood loss.

**Outcomes:**

The patient recovered well, and no abnormalities were observed during the following 2 years. Pathological results showed that this adnexal mass was a type of cavernous hemangioma arising from the infundibulopelvic vessels.

**Lessons:**

Surgical removal of the affected tissues is an aggressive treatment of choice for cavernous hemangiomas. Laparoscopic resection of infundibulopelvic hemangioma is feasible, and gynecologists are qualified for this operation, as long as damage to the iliac vessels is avoided.

## 1. Introduction

Hemangiomas are composed of several vascular proliferations. This is a common soft tissue tumor that accounts for approximately 7% of all benign tumors.^[1]^ They are found in many organs, including the skin, mucosa, liver, and brain. However, benign vascular tumors of the female reproductive organs are uncommon. Hemangiomas arising from the ovaries are relatively common.^[2]^ Hemangiomas arising from the infundibulopelvic vessels are extremely rare. To our knowledge, only 2 cases have been reported.^[3,4]^ Herein, we report a case of cavernous hemangioma, arising from the infundibulopelvic vessels in a woman who underwent surgery for an adnexal mass.

## 2. Case presentation

**Figure 1.**
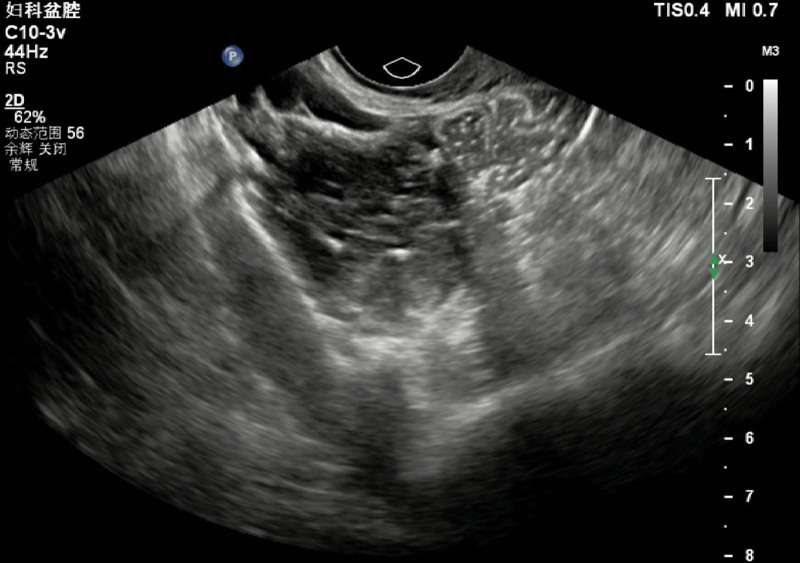
Ultrasonography pictures.

The patient was a 40-year-old woman who was admitted after physical examination revealed an ovarian mass. She had right lower abdominal pain during sexual intercourse for a month. Additionally, she had no other subjective symptoms or remarkable medical history. Physical examination revealed a palpable adnexal mass in the right pelvic cavity. Ultrasonography revealed a 4.5 × 4.0 × 5.0 cm space-occupying lesion close to the right ovary, which had many echogenic lines and calcification in the cystic cavity (Fig. 1). Thus, the ultrasonic imaging results suggest a possibility of ovarian teratoma. Serum tumor markers, such as carcinoembryonic antigen, alpha fetoprotein, carbohydrate antigen 19-9, and carbohydrate antigen 125, were within the normal range. Routine preoperative laboratory inspections (such as routine blood tests, coagulation, serum biochemistry, infectious disease screening, and ABO and RhD typing), chest radiography, and electrocardiography were normal.

**Figure 2.**
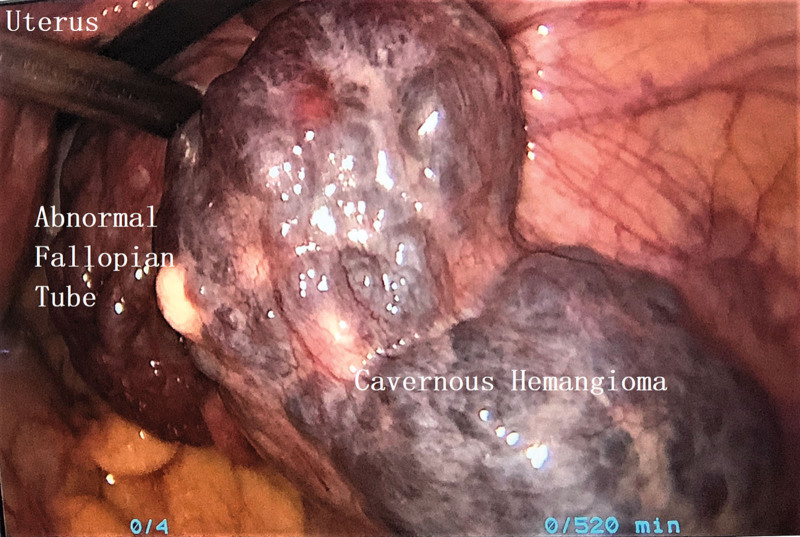
Cavernous hemangioma adhered to the pelvic wall, arising from the infundibulopelvic vessels to the mesosalpinx.

**Figure 3.**
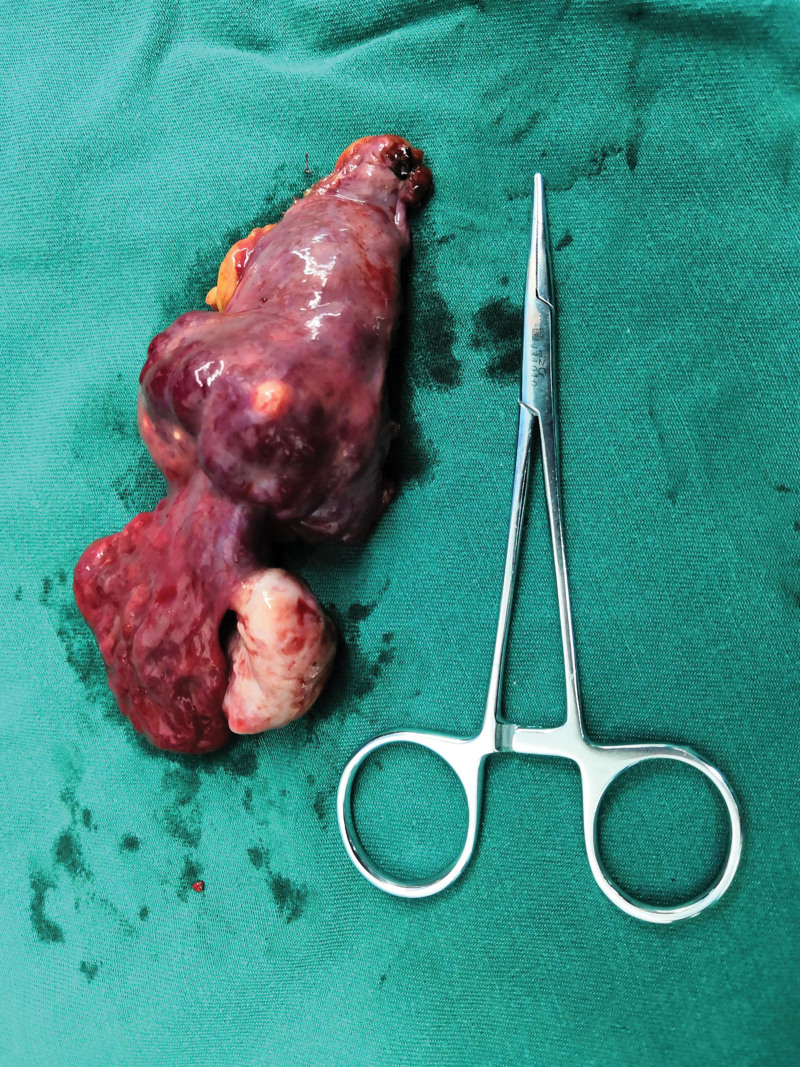
Surface of the tumor, fallopian tube, and ovary.

**Figure 4.**
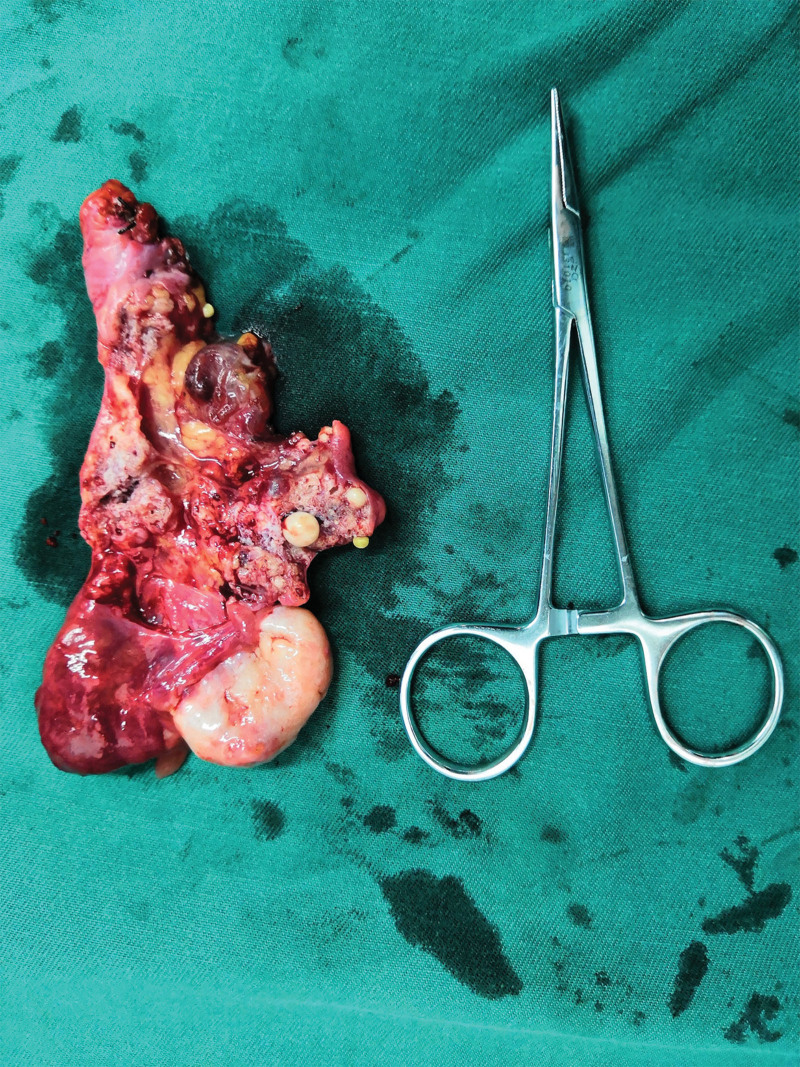
Cut surface was of honeycomb.

The patient underwent laparoscopic surgery on 20/8/2018, and the mass was a retroperitoneal hemangioma with dilated and distorted blood vessels. The mass was then carefully dissected through a peritoneal incision. When we attempted to locate the origin of the dominant vascular supply of the lesion during the dissection process, we found that it was connected to the right ovary and right infundibulopelvic vessels (Fig. 2). The right ovary appeared normal; however, the right fallopian tube was varicose. We cut off the proximal part of the infundibulopelvic vessels and proper ligament of the ovary. The hemangioma was then dissected from the surrounding retroperitoneal tissues using an ultrasonic scalpel. The operative findings indicated that there was no relationship with other muscles or vessels. We mostly used electrocoagulation to control bleeding. After complete resection, the excised specimen was extracted through a small abdominal incision (Figs. 3, 4).

**Figure 5.**
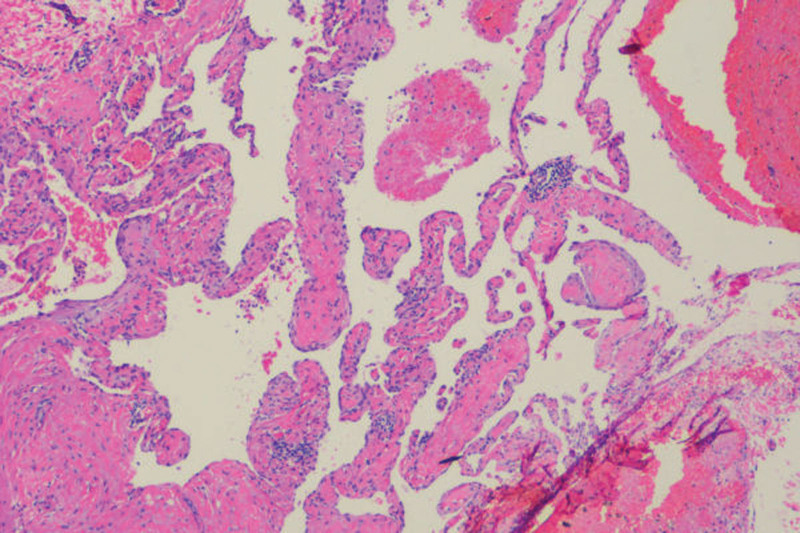
Pathological result confirmed the diagnosis of cavernous hemangioma (×100). The hemangioma was located in the infundibulopelvic vessels and salpinx. It was characterized by dilution of blood vessels, most of which were thin-walled vessels, and small vessels were thick-walled vessels.

The operation time was 90 minutes, and the overall blood loss was 50 mL. The patient made an uneventful recovery to normal health and was discharged on postoperative day 4. Pathological examination results demonstrated that the mass was a type of cavernous hemangioma, consisting of calcified nodules in homogeneously dilated and blood-filled vessels (Fig. 5). The right fallopian tube contained inhomogeneous, dilated, and blood-filled vessels.

The patient recovered well, and no abnormalities were observed during the following 2 years. All clinical datasets were collected with prior informed consent from the patient’s tutor in conformity with the Medical Ethics Committees of West China Second University Hospital of Sichuan University. Consent for publication was obtained from the patient, and a consent form was signed.

## 3. Discussion

Hemangioma is a nonfunctioning benign tumor commonly found in many organs, with a higher occurrence during infancy and childhood. Approximately 80 cases of fallopian and ovarian hemangiomas have been reported in the female reproductive system in English papers since the first report by Payne in 1869.^[5]^ Hemangioma arising from infundibulopelvic vessels is rarely found; therefore, only 2 cases have been reported (Table 1).^[3]^ The etiology of such hemangioma is unknown, and preoperatively, the differential diagnosis is rather challenging. A similar case was that of a 29-year-old woman from South Korea who was incidentally found to have retroperitoneal cavernous hemangioma, originating in the blood vessels of the infundibulopelvic ligament and was also treated by laparoscopic excision.^[3]^

**Table 1:** Clinical and pathologic findings of cases with hemangioma of infundibulopelvic vessels reported in the literature.

Authors	Year	Language	Age	Symptoms	Size (cm)	Pathologic type
Han, et al.^[4]^	2005	Chinese	36	No subjective symptom	7.2 × 5.2 × 6.2	Cavernous
Choi Y.S., Oh H.K.^[3]^	2009	English	29	No subjective symptom	5 × 4	Cavernous

Histopathologically, hemangiomas are classified into several categories, according to vessel size and wall thickness: capillary hemangioma, cavernous hemangioma (angiocavemoma), and racemosum hemangioma. The incidence of cavernous hemangiomas is lower than that of capillary hemangiomas. Clinically, cavernous hemangiomas differ from capillary hemangiomas in several essential features. Cavernous hemangiomas are usually larger and less restricted; therefore, they may infiltrate deep tissue structures more frequently. They usually do not tend to recede and may even be locally destructive by exerting pressure on adjacent tissue structures. Therefore, a large proportion of cavernous hemangiomas frequently require operative resection in contrast to capillary hemangiomas.^[1]^

In our case, the tumor was a cavernous hemangioma with thin vessel walls and diluted cavities, which implicated both infundibulopelvic and fallopian vessels. Cavernous hemangiomas are congenital defects derived from the mesoderm and consist of dilated blood vessels, including huge blood-filled spaces, which are caused by the expansion and thickening of capillary walls.^[6]^ Because of slow blood flow, dystrophic mineralization in an organizing thrombus leads to the formation of phleboliths in the mass.^[7]^ Cavernous hemangiomas have been recorded in a variety of organs, including the liver, spleen, colon, adrenal glands, bone, mediastinum, central nervous system, and other soft tissues.^[8]^ Genetic investigations have indicated that cavernous hemangiomas from the brain and retina are of autosomal dominant inheritance.^[9]^ Furthermore, the mutation in the KR1T1/CCM1 gene may lead to premature protein termination, which may facilitate the occurrence of retinal and cerebral cavernous hemangiomas.^[10]^ In the female reproductive system, cavernous hemangiomas are mostly found in the ovaries, or sometimes in the uterus, cervix, and fallopian tubes.

According to the imaging findings of this case, the differential diagnosis should include mature cystic teratoma of the ovary, vascular tumors (such as angiosarcoma, lymphangioma, and hemangioma), and other soft tissue tumors (such as gastrointestinal stromal tumor and fibroma). Our ultrasonic imaging results indicated the possibility of a mature cystic teratoma; therefore, we initially considered a teratoma of the ovary. If possible, an additional contrast-enhanced computerized tomography may help to differentiate the hemangioma from the teratoma.^[11]^ According to literature report, lymphangiomas usually do not have calcifications, and angiosarcomas do not have fatty hyperplasia, but they often show more infiltrative and invasive features in the surrounding tissues.^[12]^ Generally, gastrointestinal stromal tumors and fibromas usually have well-defined and smooth boundaries, which are homogeneous solid hypoechoic masses on ultrasound.^[3]^ They are different from this case.

## 4. Conclusions

Cavernous hemangiomas of the infundibulopelvic vessels are rarely diagnosed and are often discovered incidentally during health examination or abdominal surgery. Surgical removal of the affected tissues is an aggressive treatment of choice. Based on our operative process and literature review, we believe that retroperitoneal hemangiomas derived from the infundibulopelvic vessels can be resected successfully with low bleeding and minimal invasion by gynecologists qualified for this surgery. Further studies are needed to evaluate the long-term effectiveness of surgery.

## Author contributions

Conceptualization: Xin Tan, Hongwei Ma.

Data curation: Huiyun Tang, Qian Chen.

Visualization: Wen Zheng.

Writing – original draft: Hongwei Ma.

Writing – review and editing: Hongwei Ma.

## Acknowledgments

We thank Prof. Gang Shi and Prof. Yuedong He for their assistance with the operation. We would also like to thank Ms. Yiwen Yang for her help with English language editing.
